# ﻿Notes on Brazilian *Pachira* (Malvaceae, Bombacoideae) II: Additional typifications and new synonymies

**DOI:** 10.3897/phytokeys.186.71445

**Published:** 2021-12-06

**Authors:** Jefferson Carvalho-Sobrinho, Vania Nobuko Yoshikawa, Laurence J. Dorr

**Affiliations:** 1 Universidade Federal do Vale do São Francisco – UNIVASF, Colegiado de Ciências Biológicas, Petrolina, Pernambuco, 56300-990, Brazil Universidade Federal do Vale do São Francisco Petrolina Brazil; 2 Universidade Federal Rural de Pernambuco – UFRPE, Departamento de Ciências Florestais, Recife, Pernambuco, 52171-900, Brazil Universidade Federal Rural de Pernambuco Recife Brazil; 3 Universidade de Mogi das Cruzes – UMC, Programa de Pós-Graduação em Biotecnologia, Mogi das Cruzes, São Paulo, 08780-911, Brazil Universidade de Mogi das Cruzes Mogi das Cruzes Brazil; 4 Department of Botany, MRC-166, Smithsonian Institution, P.O. Box 37012, Washington DC 20013-7012, USA Department of Botany, Smithsonian Institution Washington DC United States of America

**Keywords:** Bombacoideae, *Bombax*, Brazil, *Carolinea*, Malvaceae, *Pachira*

## Abstract

The typification and status of the names of 14 species of *Pachira* (Malvaceae: Bombacoideae) found in Brazil are discussed, including type material from Brazil, the Guianas, Colombia, Venezuela, and cultivated in Algeria. We designate 11 lectotypes, three neotypes, and four epitypes for these names. Six names are newly considered to be synonyms of the species accepted here. The results support a forthcoming taxonomic treatment of *Pachira* for the Flora of Brazil.

## ﻿Introduction

*Pachira* Aubl. is the most species-rich genus among the 17 genera of Bombacoideae (Malvaceae) (Carvalho-Sobrinho et al. 2016) and it consists of trees distributed primarily in wet forest in northern South America (Robyns 1963; Alverson 1994; Fernández-Alonso 1998, 2003; Carvalho-Sobrinho et al. 2014). About one-hundred names have been published in *Pachira* (IPNI 2021; Tropicos 2021), but they probably represent only about 50 species in this genus. The genus was last revised by Robyns (1963). Since then, generic concepts have changed radically and a contemporary revision of *Pachira* would resolve not only questions about species richness, but also clarify evolutionary relationships and biogeography.

*Pachira* includes several Neotropical taxa originally described in other genera, including *Bombacopsis* Pittier, *Bombax* L. (Carvalho-Sobrinho and Dorr 2020), *Carolinea* L. f., *Pochota* Ram. Goyena, *Pseudobombax* Dugand, and *Rhodognaphalopsis* A. Robyns (Cuatrecasas 1954; Robyns 1963, 1967; Steyermark and Stevens 1988). During the preparation of a taxonomic treatment of *Pachira* for the Flora of Brazil 2020 project (http://floradobrasil.jbrj.gov.br), we encountered several names in *Bombax*, *Carolinea*, *Pachira*, and *Rhodognaphalopsis* that require typification and/or clarification.

Herein we discuss the typification and status of the names (and some of their synonyms) of 14 species of *Pachira* found in Brazil. Type material is from Brazil (including Brazilian material cultivated in Algeria), the Guianas, Colombia, and Venezuela. We designate 11 lectotypes, three neotypes, and four epitypes for these names. Notably, an epitype is designated here for *P.aquatica* Aubl., the type of the genus. Six names are newly considered to be synonyms of the species accepted here. Most significantly, *P.nitida* Kunth, a name often used in checklists of the Brazilian flora (see e.g., Duarte 2010; BFG [The Brazilian Flora Group] 2015), is revealed to be a synonym of *P.minor* (Sims) Hemsl. Similarly, *P.dolichocalyx* A. Robyns, previously considered to be endemic to French Guiana, is shown to be a synonym of *P.macrocalyx* (Ducke) Fern. Alonso that was described from Brazil.

## ﻿Materials and methods

Protologues of names of *Pachira* taxa found in Brazil (and their basionyms) were examined along with relevant revisionary and floristic literature in order to determine what constituted original material, the identities of these taxa, and to establish whether these names had been typified. The specimens cited as types were either examined by us in person or via digital proxies. Herbarium acronyms for these specimens follow Thiers (2021 continuously updated). The notation “F neg. no.” refers to the “Berlin Negatives” of the Field Museum (F), a unique type-photographic collection of European herbaria that J. Francis Macbride began assembling in 1929 (https://www.fieldmuseum.org/node/5186). These photographic images frequently are the only records of types or original material that was destroyed during WWII and these images are commonly found in northern hemisphere herbaria.

For the sake of brevity, we do not provide complete synonymies for the *Pachira* taxa that we discuss, but rather list only names that have not been, or were previously incorrectly, typified. In a few instances, we include names that were typified earlier, but whose citations require clarification. We also list names of taxa that are considered here to be new synonyms irrespective of their typification status.

## ﻿Typification

### ﻿ Pachira aquatica

Taxon classificationPlantaeMalvalesMalvaceae

Aubl., Hist. Pl. Guiane 2: 726, tt. 291, 292. 1775.

F1B99D7C-E4C5-5693-8EE9-9E9A874181BF

Figs 1 , 2A

Carolinea princeps L. f., Suppl. Pl.: 314. 1782 [1781], nom. illeg. superfl. Pachiracarolinea Dum. Cours., Bot. Cult., ed. 1, 3: 84. 1802, nom. illeg. superfl. Bombaxaquaticum (Aubl.) K. Schum., in Engler & Prantl, Nat. Pflanzenfam. 3(6): 62. 1895.
Carolinea pompalis Moc. & Sessé ex DC., Prodr. 1: 478. 1824, nom. nud., pro syn.
Pachira grandiflora Tussac, Fl. Antill. 4: 12, tt. 3, 4. 1827. Carolineagrandiflora (Tussac) Spach, Hist. Nat. Vég. 14: 206. 1847 [1848]. Type: “Antilles.” Lectotype, designated by Yoshikawa et al. (in press): Tussac (1827, t. 3).
Carolinea macrocarpa Schltdl. & Cham., Linnaea 6: 423. 1831. Pachiramacrocarpa (Schltdl. & Cham.) Walp., Repert. Bot. Syst. 1(2): 329. 1842. Pachiralongifolia Hook., Bot. Mag., ser. 3, 6: t. 4549. 1850, nom. illeg. superfl. Bombaxmacrocarpum (Schltdl. & Cham.) K. Schum., in Engler & Prantl, Nat. Pflanzenfam. 3(6): 62. 1895. Type: Mexico. Veracruz, Papantla, Feb 1829 (lf, fl bud), F. Deppe & C.J.W. Schiede 1312 (lectotype, designated by Yoshikawa et al. (in press): BM barcode 000645670 [s.n.]; isolectotypes: HAL barcode 0098398, HAL barcode 0128027 (2 sheets), HAL barcode 0128028 [s.n.] (2 sheets), LE n.v., W n.v.).
Carolinea lovisa-carolina L. ex B.D. Jacks., Index Linn. Herb.: 52. 1912, nom. nud.

#### Type.

French Guiana. Cayenne, 1775 (lf), *F. Aublet s.n.* (lectotype, as “holotype,” designated by Robyns 1963, pp. 238, 242: BM barcode 000645671). Epitype: French Guiana. Tour de l’Ile River, 0–10 m, 04°47' 36"N, 052°22' 38"W, 18 Oct 1991 (lf, fl), *S.A. Mori* et al. *22126* (epitype, designated here: US barcode 00636701; isoepitypes: CAY barcode 155330, NY barcode 402480).

#### Note.

Yoshikawa et al. (in press) typified several names that are synonyms of *Pachiraaquatica*, but omitted mention of *nomina nuda*. Despite their lack of nomenclatural standing, these *nomina nuda* associated with *P.aquatica* do appear in standard indices (e.g., IPNI 2021; Tropicos 2021) and we include them to clarify their identification. Likewise, we discuss two names typified by Yoshikawa et al. (in press) in order to clarify their synonymies and bibliographic citations that are confused in standard indices.

**Figure 1.**
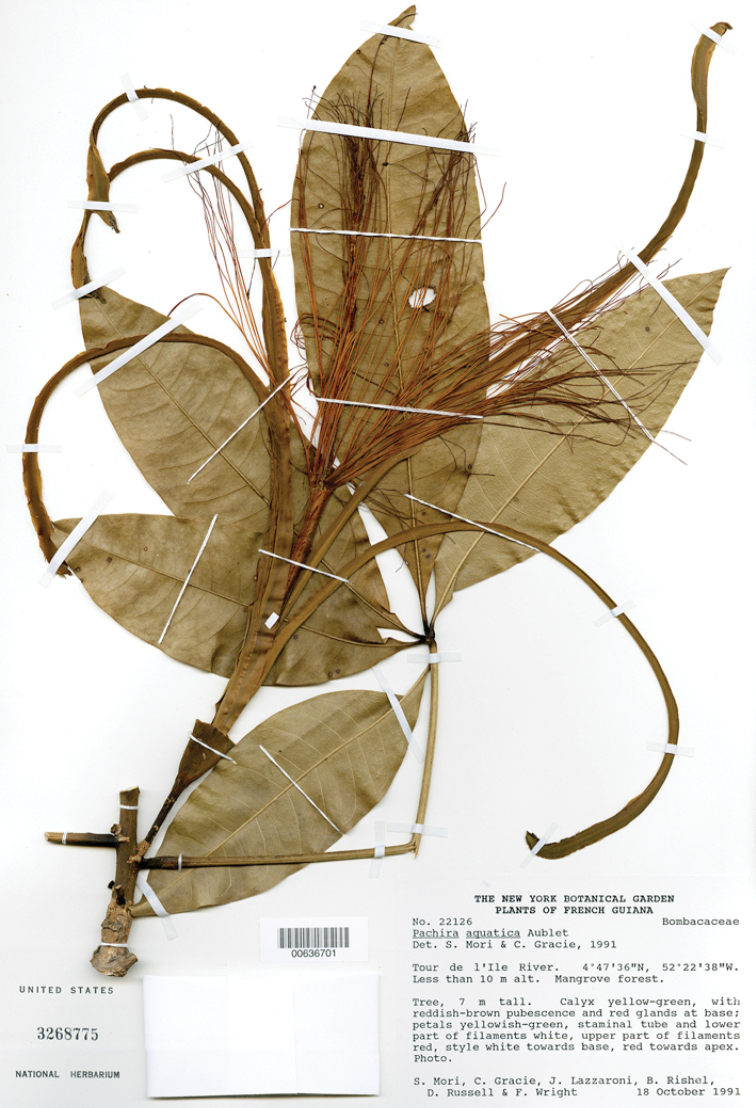
Epitype of *Pachiraaquatica* Aubl. (US barcode 00636701).

The lectotype of *Pachiraaquatica* consists solely of detached leaflets and a sterile twig. Consequently, a flowering collection from French Guiana is designated here as an epitype (Fig. 1).

“*Carolineapompalis fl. mex. ic. ined.*” was cited originally as a synonym of *C.minor* Sims (≡ *Pachiraminor* (Sims) Hemsl.). Robyns (1963) did not agree with this interpretation and placed the *nomen nudum* among his “*Species dubiae incertae sedis*.” McVaugh (2000, p. 88) discussed the sources of the material that de Candolle (1824) examined, which included a plate in G (see F neg. no. 30513) and presumably another illustration now in the Torner Collection (Hunt Institute for Botanical Documentation accession No. 6331.0864; see also No. 6331.1977). These images depict *P.aquatica*, which occurs in Mexico and South America. *Pachiraminor* is restricted to South America, which was not visited by the Sessé and Mociño Expedition.

Several indices (e.g., IPNI 2021; Tropicos 2021) state incorrectly that the combination *Carolineagrandiflora* (Tussac) Spach was made in 1834, but Spach (1834, p. 426) did not then definitely associate the epithet “*grandiflora*” with the genus *Carolinea* stating simply “Carolinéa de Tussac. – *Pachiragrandiflora* Tussac” (see Turland et al. 2018; Art. 35.2). The combination was made in 1847 in an index to the larger work when Spach (1847, p. 206) wrote “[Carolinea] grandiflora, Tuss. .. [Vol.] 3 [Pag.] 426": the volume and page numbers providing an indirect reference to the basionym (see Turland et al. 2018; Art. 41.3).

*Carolineamacrocarpa* was described from Mexican material cultivated in Berlin. The protologue gives the type locality as “*Ad ripas fluminum et rivulorum Papantlensium et Tecolutensium*. *Papantlae*.” Robyns (1963, p. 239) selected “*Schiede* et *Deppe* 1312" as “typus” and he cited specimens at BM, LE and W, but not HAL. The labels on the HAL specimens, however, suggest the collecting combination may have been “Deppe & Schiede” and not “Schiede & Deppe.”

*Pachiralongifolia* is a superfluous name for *P.macrocarpa* (≡ *Carolineamacrocarpa*). The epithet of this superfluous name is often miscited as “*longiflora*” (see e.g., Robyns 1963; Alverson in Berry et al. 1997), which is perhaps understandable given that Hooker (1850) called it the “Long-flowered *Pachira*.”

### ﻿ Pachira calophylla

Taxon classificationPlantaeMalvalesMalvaceae

(K. Schum.) Fern. Alonso, Anales Jard. Bot. Madrid 56: 308. 1998.

FA2CE904-939D-5514-8DD3-AC3E8FC8DB22

Bombax calophyllum K. Schum., in Martius, Fl. Bras. 12(3): 227. 1886. Bombacopsiscalophylla (K. Schum.) A. Robyns, Bull. Jard. Bot. État Bruxelles 33: 201. 1963.
Bombax stenopodum Ulbr., Notizbl. K. Bot. Gart. Mus. Berlin 6: 55. 1914. Type: Brazil. Rio de Janeiro, s.d. (lf, fl), *L. Riedel s.n.* (neotype, designated here: LE barcode 00003676; isoneotype: LE barcode 00003677; possible isoneotype: S-PLE-E4208 n.v.).

#### Type.

Brazil. Rio de Janeiro, s.d. (lf, fl), *L. Riedel s.n.* (lectotype, designated here: LE barcode 00003676; isolectotype: LE barcode 00003677; possible isolectotype: S-PLE-E4208 n.v.).

#### Note.

Robyns (1963, p. 203) designated “Riedel s.n. (LE)” as lectotype of *Bombaxcalophyllum*. When Fernández-Alonso (1998) made the combination in *Pachira*, he accepted Robyn’s type designation, but stated that he had not seen the lectotype. Robyns annotated the two Riedel specimens in St. Petersburg (LE) cited above as “*Bombacopsiscalophylla* (K. Schum.) A. Robyns, *comb. nov.*” and wrote “*lectotypus*” on the one specimen that has a handwritten label indicating the type locality as “*Brasiliae*: *R. Janeiro*,” locality information that matches the protologue. The ICN (Turland et al. 2018; Art. 7.10) requires that a type designation be effectively published and the mere annotation of a herbarium sheet does not meet this requirement. Our lectotypification (second step; see Turland et al. 2018; Art. 9.17) narrows the type to a single element, a gathering that has loose leaves and a fragmented flower (calyx, ovary, and androecium). The isolectotype is sterile.

The protologue of *Bombaxstenopodum* cites a single collection, “E. Ule n. 4631,” which was deposited in Berlin (B†; see F neg. no. 9545). Inasmuch as the Berlin specimen was destroyed in WWII and no duplicate material has been found, we designate a neotype for this name. Our choice makes this name an obligate synonym of *Pachiracalophylla*.

### ﻿ Pachira duckei

Taxon classificationPlantaeMalvalesMalvaceae

(A. Robyns) Fern. Alonso, Anales Jard. Bot. Madrid 56: 310. 1998.

208F3CC0-0C2D-58BA-8C31-BB5AAD2A8537

Fig. 2B

Rhodognaphalopsis duckei A. Robyns, Bull. Jard. Bot. État Bruxelles 33: 275, fig. 14. 1963.
Rhodognaphalopsis duckei var. obtusifolia A. Robyns, Bull. Jard. Bot. État Bruxelles 33: 278. 1963, ***syn. nov.*** Type: Brazil. Amazonas, Marmellos, Rio Madeira, Mar 1902 (lf, fl), *E. Ule 6077* (holotype: G barcode 00177546; isotypes: K barcode 000382340, L barcode 0012900, RB barcode 00534490, RB barcode 00534520).

#### Type.

Brazil. Amazonas, Manaus, Igarapé da Raiz, 30 Aug 1946 (lf, fl), *A. Ducke 310* (IIa. col.) (holotype: MG barcode 018577 [= MG n.° 18.577]; isotypes: IAC 13840, IAN, SP barcode SP 003366).

**Figure 2.**
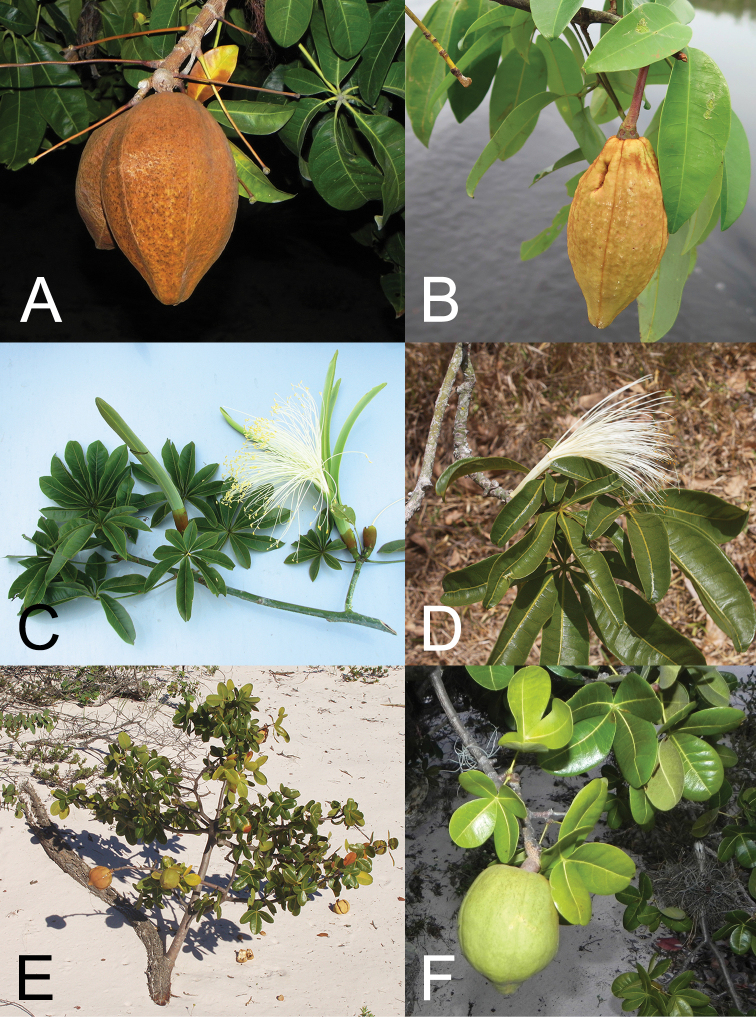
*Pachira* species **A***P.aquatica* fruit **B***P.duckei* fruit **C***P.endecaphylla* leaves and flower **D***P.glabra* leaves and flower **E***P.retusa* habit **F***P.retusa* leaves and fruit. Photographs **A, D** J.G. Carvalho-Sobrinho **B** C.E. Zartman **C** B.A.S. Pereira **E** M.C. Machado **F** L.P. Queiroz.

#### Note.

When Robyns (1963) described *Rhodognaphalopsisduckei* and R.d.var.obtusifolia he had very little material available. In fact, he cited fewer than a dozen collections for both taxa. The characters that he used to distinguish var.obtusifolia from the nominate variety were subtle and overlap with specimens he cited as paratypes of the latter. We cannot distinguish the obtuse leaflet bases of *Ducke s.n.* [RB 23484] (see e.g., K barcode 000913930), a paratype of *R.duckei*, from those of *Ule 6077* (see e.g., K barcode 000382340), the type of R.d.var.obtusifolia. For this reason and because the two taxa lack discrete ranges, we propose these names should be treated as synonyms.

### ﻿ Pachira endecaphylla

Taxon classificationPlantaeMalvalesMalvaceae

(Vell.) Carv.-Sobr., Taxon 62: 816. 2013.

5025B785-DBAD-5BC9-93AD-A2A13281534F

Fig. 2C

Bombax endecaphyllum Vell., Fl. Flumin.: 288. 1829 [1825], Ibid., Fl. Flumin. Icones 7: t. 50. 1831 [1827]. Pseudobombaxendecaphyllum (Vell.) A. Robyns, Bull. Jard. Bot. État Bruxelles 33: 60. 1963.
Pachira stenopetala Casar., Nov. Stirp. Bras.: 21. 1842. Bombaxstenopetalum (Casar.) K. Schum., in Martius, Fl. Bras. 12(3): 226, t. 45. 1886. Bombacopsisstenopetala (Casar.) A. Robyns, Bull. Jard. Bot. État Bruxelles 33: 221. 1963. Type: Brazil. “Occurrit passim prope Rio de Janeiro,” s.d. [Nov 1838] (fl, fl), *Casaretto Herb. No. 581* (lectotype, designated by Delprete et al. 2019, p. 802): TO [sheet No. 2] n.v.).
Pachira elegans Hooibr. ex Planch., Hort. Donat. 23. 1858, ***syn. nov.*** Type: Hort. Paris, 1854 [lf], without collector (lectotype, designated here: MPU barcode 761966).

#### Type.

Lectotype, designated here: “Monad. Polyand. *BOMBAX endecaphyllum* tab. 50" (original pen and ink drawing for the *Flora Fluminensis* preserved in the Manuscript Section, Biblioteca Nacional do Brasil, Rio de Janeiro [cat. no.: mss1198656_054]). Epitype: Brazil. Espírito Santo, Santa Leopoldina, Morro Agudo, propr. Dona Maria, 20°05'51"S, 40°25'44"W, 28 Jan 2008 (lf, fl), *V. Demuner* et al. *4907* (epitype, designated here: HUEFS; isoepitypes: MBML, HVASF n.v.).

#### Note.

Carvalho-Sobrinho et al. (2013) accepted the “lectotype” designation of Robyns (1963, p. 61) for the name *Bombaxendecaphyllum*, but Robyns selected one of the plates published in the *Flora Fluminensis Icones* (1831), which is not original material and thus his designation can be superseded (Turland et al. 2018; Art. 9.19(a)). The published Vellozo plates were prepared and issued posthumously and there is no evidence that Vellozo ever saw them. Original pen and ink drawings that serve as the basis for these published plates, however, are archived in the Biblioteca Nacional do Brasil, Rio de Janeiro (see http://objdigital.bn.br/acervo_digital/div_manuscritos/mss1198656/mss1198656.htm).

Delprete et al. (2019) re-examined the typification of *Pachirastenopetala* and determined that the holotype cited by Carvalho-Sobrinho et al. (2013) did not agree with the protologue and they further established that the only original material available is the specimen in the Casaretto Herbarium (TO) that they designated as lectotype.

The specimen designated here as lectotype of *Pachiraelegans* is labeled “Pachira?elegans, Daniel” in Planchon’s hand. It was collected in 1854 before the name was published and it is now in the Herbier Planchon (MPU). It therefore can be considered original material (see Turland et al. 2018; Art. 9.4(a)) even though it is labeled “Hort. Paris” and not Hortus Donatensis. In addition, although the specimen is sterile the description of setiform mucros terminating leaflet apices matches the physical specimen. The protologue also cites “Brésil. Rio de Janeiro, *Gaudich*. in Herb. Mus. Par.,” which almost certainly is a reference to two collections made by Ch. Gaudichaud that have leaves that are superficially similar to *Pachiraendecaphylla*. These collections (*Gaudichaud 945* and *945bis*) were discussed in detail by Carvalho-Sobrinho et al. (2013) who considered them both to be mixtures and who identified their flowers as Pseudobombaxgrandiflorumvar.majus A. Robyns and their leaves as *Jacaratiaspinosa* (Aubl.) A. DC. (Caricaceae). Since flowers are not mentioned in the protologue of *Pachiraelegans* and the leaflets of the Gaudichaud collections do not have setiform mucros (and are from a different plant family), we do not think either Gaudichaud collection is suitable as a lectotype. Planchon (1858, p. 232) identified “Daniel” as the horticulturist Daniel Hooibrenk (1813–1895).

### ﻿ Pachira glabra

Taxon classificationPlantaeMalvalesMalvaceae

Pasq., Rendiconto Reale Accad. Sci. Fis. 7: 18. 1868.

A0D7450C-6CF9-5518-B310-A7DEDB1B4E4E

Fig. 2D

Bombax glabrum (Pasq.) A. Robyns, Bull. Jard. Bot. État Bruxelles 30: 474. 1960. Bombacopsisglabra (Pasq.) A. Robyns, Bull. Jard. Bot. État Bruxelles 33: 207. 1963. Pochotaglabra (Pasq.) Bullock, Kew Bull. 20: 528. 1966.
Pachira oleagina Decne., Ann. Gén. Hort. 23: 49. 1881? [1880]. Bombaxoleaginum (Decne.) A. Robyns, Bull. Jard. Bot. État Bruxelles 29: 26. 1959. Type: Algeria. Jardin du Hamma, près d’Algers, 1879 (lf, fl), *Ch. Rivière s.n.* (neotype, designated here: P barcode 02286303; isoneotypes: P barcode 02286301, P barcode 02286302 [= F neg. no. 35351]).
Bombax anisophyllum Buxb., Oesterr. Bot. Z. 73: 121. 1924. Type: Brazil. Paraná, Antonina, 1904 (lf, fl), *M. Wacket s.n.* (lectotype, designated here: WU 0073137; isolectotypes: W barcode 19240009283, WU 0073138).

#### Type.

Algeria. Jardin du Hamma, près d’Algers, 1879 (lf, fl), *Ch. Rivière s.n.* (neotype, designated here: P barcode 02286303; isoneotypes: P barcode 02286301, P barcode 02286302 [= F neg. no. 35351]).

#### Note.

Robyns (1960) established that there is no extant original material of *Pachiraglabra*. He then neotypified (first step) the names *P.glabra* and *P.oleaginea* with the same collection (i.e., “Rivière s.n.”). This collection is represented by three sheets in Paris (P), all three of which were labeled “Neotypus” by Robyns. The three sheets are not marked as being part of the same specimen and we interpret them as duplicates of a single gathering (see Turland et al. 2018; Art. 8.3). Accordingly, we designate here one of the three sheets as the neotype (second step) for the names *P.glabra* and *P.oleaginea*. One of the isoneotypes (P barcode 02286301) has a faint pencil sketch of the fruit that was labeled by Decaisne “fructus maturus magnif. nat.”

The labels on the neotype and one of the two isoneotypes indicate that the collection was made by “Cl. Ch. Rivière” where “Cl.” is Latin for *clarissimus* (i.e., distinguished). The latter label also has the initial “A.” added below this name, presumably a reference to A. Rivière who, however, was deceased before the collection was made. The horticulturists and brothers [Marie] Auguste Rivière (1821–1877) and Charles Marie Rivière (b. 1845) were successive directors of the Jardin du Hamma, the latter succeeding the former (Stafleu and Cowan 1983).

### ﻿ Pachira humilis

Taxon classificationPlantaeMalvalesMalvaceae

Spruce ex Decne., Ann. Gén. Hort. 23: 52. 1881? [1880].

1558AE75-D14F-5582-98DB-63BAC8219C51

Bombax humilis (Spruce ex Decne.) K. Schum., in Martius, Fl. Bras. 12(3): 224. 1886. Rhodognaphalopsishumilis (Spruce ex Decne.) A. Robyns, Bull. Jard. Bot. État Bruxelles 33: 294. 1963. Pochotahumilis (Spruce ex Decne.) Steyerm. & W.D. Stevens, Ann. Missouri Bot. Gard. 75: 397. 1988.
Pachira humilis Spruce ex Benth., J. Proc. Linn. Soc., Bot. 6: 108. 1862, nom. nud.

#### Type.

Venezuela. Amazonas, [prope] San Carlos, [ad Rio Negro], Nov 1853 (lf, fl), *R. Spruce 3135* (lectotype, as “holotypus,” designated by Robyns 1963, p. 296): K barcode 000382334 [handwritten label]; isolectotypes: BM barcode 000645664 [1853–54], BR barcode 0000006961398 [1853–54], E barcode 00285199 [1853–54; handwritten label], F barcode F0052164F [s.d.; fragment], GH barcode 00071941 [s.d.], LD barcode 730579 [1853–54], NY barcode 00133523 [1853–54], P barcode 02285964 [locality illegible; Nov 1854 (sic)], S-PLE-E4502 n.v., TCD barcode 0000900 [1853–54], W barcode 18890017581 [1853–54; handwritten label], W barcode 18890123939 [1853–54; handwritten label]).

#### Note.

A collection, “Pachirahumilis, Spruce, *Mss.* (exsicc. 3135),” is cited in the protologue, but Decaisne (1881) gives no explicit indication as to where he examined material. The description of this species (and others in the same article) ends with the phrase “H. Mus. Par.” Presumably this is a reference to material in the herbarium and not the gardens (“hortus”) in Paris since Ch. Rivière, Director of the Jardin du Hamma in Algeria is acknowledged by Decaisne for providing him with fresh flowers and fruit of *Pachira* species.

The synonymy and authorship of *Pachirahumilis* Spruce ex Decne. have been confused because of the mistaken belief that Bentham (1862) transferred the species to *Bombax*. Bentham cited the unpublished name “*Pachirahumilis*, Spruce” in a discussion regarding the distinction between *Bombax* and *Pachira*, but he did not definitely associate the final epithet “*humilis*” with *Bombax* (see Turland et al. 2018; Art. 35.2). Schumann (1886, p. 224) was the first to make the combination in *Bombax*, albeit inadvertently. He recognized “*Bombaxhumile*, Benth.” and he cited the basionym when he wrote “*Pachirahumilis Spruce Msc.! in schedulis*; *Dcne. Miscell. bot. 1880*, *p. 10*.” The subordinate phrase is a reference to “*Miscellaneabotanica*,” which reprints Decaisne’s (1881) article on *Bombax* and *Pachira* including the original description of *P.humilis*.

### ﻿ Pachira insignis

Taxon classificationPlantaeMalvalesMalvaceae

(Sw.) Sw. ex Savigny, in Lamarck, Encycl. 4: 690. 1798.

268670BE-A904-58B6-B30A-B64786AEB9B0

Carolinea insignis Sw., Prodr. 101. 1788. Pachiraloddigesii Decne., Ann. Gén. Hort. 23: 51. 1881? [1880], nom. illeg. superfl. Bombaxinsigne (Sw.) K. Schum., in Engler & Prantl, Nat. Pflanzenfam. 3(6): 62. 1895, non Wall., Pl. Asiat. Rar. 1: 71, tt. 79, 80. 1830. Bombaxspectabile Ulbr., Bot. Jahrb. Syst. 49: 544. 1913, nom. nov.
Carolinea affinis Mart., Nov. Gen. Sp. Pl. 1: 85. 1826 [1824]. Pachiraaffinis (Mart.) Decne., Ann. Gén. Hort. 23: 52. 1881? [1880]. Bombaxaffine (Mart.) Ducke, Arch. Jard. Bot. Rio de Janeiro 5: 162. 1930. Type: Brazil. Pará, Habitat in aquaticis Archipelagi Paraënsis, s.d. (lf, fl), *C.F.P. von Martius s.n.* (lectotype, designated here: M barcode 0211673; isolectotype: M barcode 0211672).
Pachira macrantha Spruce ex Decne., Ann. Gén. Hort. 23: 47. 1881? [1880], nom. nud., pro syn.
Pachira spruceana Decne, Ann. Gén. Hort. 23: 46. 1881? [1880]. Bombaxspruceanum (Decne.) Ducke, Arch. Jard. Bot. Rio de Janeiro 4: 126. 1925. Type: Brazil. Amazonas, Panure ad Rio Uaupes, Oct 1852-Jan 1853 (lf, fl), *R. Spruce 2884* (lectotype, designated here: P barcode 04694521 [= F neg. no. 35353]; isolectotypes: BM barcode 000645672, BR barcode 0000006960704, BR barcode 0000006962050, E barcode 00285198, F n.v., G n.v., K barcode 000382357, K barcode 000382358, LD barcode 1758563, LD barcode 1758627, LE n.v., NY n.v., P barcode 06715161, P barcode 06623110, P barcode 06623111, P barcode 04694522, RB barcode 00059374 (2 sheets), W n.v.).

#### Type.

Brazil. Amazonas, Paraná da Eva, Rio Amazonas, abaixo de Manaus, 27 Mar 1943 (lf, fl), *A. Ducke 1211* (neotype, designated by Robyns 1963, pp. 250, 252: MO barcode 309160; isoneotypes: K barcode 000382356, MG n.v., NY n.v., R barcode 000055277, R barcode 000055277, R barcode 000055277a, S-PLE-E4290 n.v., S-PLE-E4291 n.v., S-PLE-E4292 n.v., US barcode 00101951, US barcode 00901732, US barcode 00901733).

#### Note.

*Pachiraloddigesii* is an illegitimate renaming of *Carolineainsignis* Sw. In his protologue, Decaisne (1881) cites “*Carolineainsignis*, Lodd., *Bot. Cab.*, 1004, (*non Swarts*).” The “name” ascribed to Loddiges, however, has no standing. Loddiges (1825) simply illustrated *C.insignis* Sw. without explicitly stating that he was illustrating the species previously described by Swartz. Robyns (1963, p. 250) designated a lectotype (as “*holotypus*”) for *P.loddigesii*, but this was incorrect since the name is typified automatically by the type of the name which ought to have been adopted under the rules (Turland et al. 2018; Art. 7.5). Similarly, Turner (2016, p. 1115) argued that “*Carolineainsignis* G. Lodd.” is a valid name, even though he noted that Loddiges cited Swartz in his description and thus provided an indirect reference to *C.insignis* Sw. Turner’s lectotypification of this “name” incorrectly attributed to Loddiges also is unnecessary.

A lectotype (second step) is designated here for the name *Carolineaaffinis* because although Robyns (1963, p. 251) stated the “*holotypus*” was in Munich (M) there are two sheets in that herbarium. Robyns annotated both sheets as *Pachirainsignis*, but did not indicate that either was type material. The specimen with label data that matches the type locality given in the protologue is designated here as the lectotype.

We also designate here a lectotype for *Pachiraspruceana*. Neither Ducke (1925) nor Robyns (1963) selected one for this name. We have chosen a specimen deposited at Paris (P) that has handwriting on Spruce’s label that matches the type locality and collecting date (“Prope Panure ad rio Uaupes, Oct 1852-Jan 1853”) cited in the protologue.

*Bombaxspectabile* was proposed as a replacement name for *B.insigne* (Sw.) K. Schum., which is a later homonym of *B.insigne* Wall. *Bombaxinsigne* Wall. is a Paleotropical species found in India, south-central China, and south-east Asia.

### ﻿ Pachira macrocalyx

Taxon classificationPlantaeMalvalesMalvaceae

(Ducke) Fern. Alonso, Anales Jard. Bot. Madrid 56: 310. 1998.

E864A0D9-2FDE-58A1-92B3-894165E938C2

Bombax macrocalyx Ducke, Arch. Jard. Bot. Rio de Janeiro 4: 124. 1925. Bombacopsismacrocalyx (Ducke) A. Robyns, Bull. Jard. Bot. État 33: 203. 1963.
Pachira dolichocalyx A. Robyns, Bull. Jard. Bot. Belg. 58: 535, fig. 1988, **syn. nov.** Type: French Guiana. Piste de St. Elie, km 16, à proximité de nos carbets bota [Sinnamary], 28 May 1980 (fl, fr), *M.F. Prévost 840* (holotype: CAY [now P barcode 0007210]; isotypes: P barcode 00077211, P barcode 05273658, U barcode 0000784, U barcode 0000785).

#### Type.

Brazil. Pará, Rio Xingú, margem do Rio Tucuruhy (curso superior), 24 Aug 1919 (lf, fl), *A. Ducke s.n.* [RB 11417] (holotype: RB barcode 00534493; isotypes: B† [= F neg. no. 9536], S-PLE-E4252 n.v., S-PLE-E4253 n.v., S-R-11282, U barcode 0000771).

#### Note.

When Robyns (1988) described *Pachiradolichocalyx* he compared it to *P.aquatica* and *P.insignis*, which according to his concept of the Bombacoideae were the only two species that comprised *Pachira*. We assume he failed to compare the material to *Bombacopsismacrocalyx* because he considered *Bombacopsis* to be a different genus.

In the protologue of *Pachiradolichocalyx*, the petals are described as greenish outside and reddish-purple inside “*in vivo*.” This information could only have come from whatever may have been inferred from examining the pressed and dried material and information on the type label, which simply states that the petals are wine red (“rouges lie de vin”). Robyns (1963, p. 204) described the petals of *P.macrocalyx* (as *Bombacopsismacrocalyx*) as pale yellow outside and whitish-puberulent inside. Yet many specimen labels of *P.macrocalyx* report red petals. Additionally, staminal-tube length, fruit and seed morphology (mainly dimensions), and habitat are similar for both species supporting our decision to consider them synonymous.

### ﻿ Pachira minor

Taxon classificationPlantaeMalvalesMalvaceae

(Sims) Hemsl., Biol. Cent.-Amer., Bot. 1: 124. 1879.

6A1D473C-A29D-549D-97D1-CAF6A87E50D6

Carolinea minor Sims, Bot. Mag. 34: t. 1412. 1811. Bombaxminus (Sims) Ducke, Arch. Jard. Bot. Rio de Janeiro 6: 65. 1933. Rhodognaphalopsisminor (Sims) A. Robyns, Bull. Jard. Bot. État Bruxelles 33: 278. 1963. Pochotaminor (Sims) Steyerm. & W.D. Stevens, Ann. Missouri Bot. Gard. 75: 397. 1988.
Pachira nitida Kunth, in H.B.K., Nov. Gen. Sp. (quarto ed.) 5: 302. 1822 [1821], Ibid. (folio ed.) 5: 235. 1822 [1821], ***syn. nov.***Rhodognaphalopsisnitida (Kunth) A. Robyns, Bull. Jard. Bot. État Bruxelles 33: 282. 1963. Pochotanitida (Kunth) Steyerm. & W.D. Stevens, Ann. Missouri Bot. Gard. 75: 397. 1988. Type: Venezuela. Amazonas, Caño de Pimichin, s.d. (fl), *F.W.H.A. von Humboldt & A.J.A. Bonpland 987* (lectotype, as “*holotypus*,” designated by Robyns 1963, p. 284): P barcode 00679764 [= F neg. No. 35354]; isolectotype: P barcode 04694524).

#### Type.

Lectotype, designated here: Sims (1811, t. 1412). Epitype: Guyana. From the interior woods of Guiana, s.d. (lf, fl), *Alex. Anderson s.n.* (epitype, designated here: BM barcode 000645662).

#### Note.

In the protologue of *Carolineaminor*, Sims (1811) states that he received this plant from “Messrs. Loddiges and Sons, under the name of *Bombax Carolinoides*, an appellation given it by Dr. Anderson of the Botanic Garden at St. Vincent’s.” Thus, original material that can be considered for a lectotype is either a cultivated specimen (or specimens) from Loddiges nursery in Hackney (now London) or the plate (t. 1412) illustrating *Carolineaminor*. Robyns (1963, p. 280) stated that the “*holotypus*” was at BM (“*Anderson* (*321* ?) (BM)”), but while it is clear that Anderson conveyed seed to Loddiges, Sims’s description was not based on Anderson’s wild-collected herbarium material and such specimens are not original material. Robyn’s “*holotypus*” cannot therefore be corrected to neotype (see Turland et al. 2018; Art. 9.10).

Robyns (1963, p. 284) effectively designated a lectotype for the name *Pachiranitida* when he stated that a collection from Caño de Pimichin made by F.W.H.A. von Humboldt and A.J.A. Bonpland was the “*holotypus*.” The handwriting on the label of this specimen (P barcode 00679764) is that of Kunth (see Stauffer et al. 2012) and the fragmentary nature of the flower agrees with statements in the protologue (here translated) that declare “Only fragments available to us. A description of a flower from the label of Bonpland.” The handwriting on the label of the isolectotype (P barcode 04694524) is that of Bonpland (see Stauffer et al. 2012). This specimen was originally identified as *Carolineaprinceps* L.f. (≡ *P.aquatica*), which also is noted in the protologue where *P.aquatica* is listed as a synonym of *P.nitida* with doubt. The morphology of the leaves (leaflet shape, venation, and strongly raised midribs on abaxial surfaces) and of the flower (calyx shape and dimensions and stamen length) of the type of *P.nitida* matches that of the type of *P.minor*.

### ﻿ Pachira nervosa

Taxon classificationPlantaeMalvalesMalvaceae

(Uittien) Fern. Alonso, Anales Jard. Bot. Madrid 56: 310.1998.

60F04439-4D24-5551-82D9-C8A87F772A0F

Bombax nervosum Uittien, Recueil Trav. Bot. Néerl. 22: 364. 1925. Bombacopsisnervosa (Uittien) A. Robyns, Bull. Jard. État Bruxelles 33: 199. 1963.

#### Type.

Suriname. Boschreserve (forest reserve), Sectie O, Boomnummer (Tree Number) 628, 8 May 1910 [sic, 1916 in protologue and on field ticket] (lf), *Forestry Bureau 1901* (lectotype, designated by Robyns 1963, p. 201: U barcode 0000772). Epitype: Brazil. Manaus, 1 Oct 1946 (lf, fl, fr), *A. Ducke 2001* (epitype, designated here: US barcode 01226557; isoepitypes: A n.v., IAN 20135, MG n.v., NY barcode 01539149, RB barcode 00054274, RB barcode 00059756, RB barcode 00775585, S-PLE-E4214 s.n.).

#### Note.

Robyns (1963, p. 201) designated “For. Bur. 1901 (U)” as the lectotype of *Bombaxnervosum*. He annotated the specimen as “*Bombacopsisnervosa* (Uitt.) A. Robyns, *comb. nov.*” and as “*lectotypus*!” Fernández-Alonso (1998, p. 310) stated that he had not seen the lectotype when he transferred *Bombaxnervosum* to *Pachira*. The lectotype of *B.nervosum*, however, is sterile and to avoid any ambiguity about its identity, an epitype is herein selected. The epitype has flowers, a determination label in Robyns’s hand, and it was cited in his revision (Robyns 1963, p. 201).

### ﻿ Pachira obtusa

Taxon classificationPlantaeMalvalesMalvaceae

Spruce ex K. Schum., in Martius, Fl. Bras. 12(3): 232. 1886.

FEB24F3F-8F0F-5BFB-92A7-44CB97F14D56

Bombax obtusum (Spruce ex K. Schum.) Bakh., Bull. Jard. Bot. Buitenzorg, sér. 3, 6: 181. 1924.
Bombax poissonianum K. Schum., in Martius, Fl. Bras. 12(3): 225. 1886, **syn. nov.** Type: Brazil. Rio Negro, s.d. (lf, fl), *sine collector* (holotype: P barcode 02285965 [= F neg. No. 35362]).
Bombax utiarityi Hoehne, Arch. Bot. São Paulo 1: 567, t. 10. 1927, ***syn. nov.***Pachirautiarityi (Hoehne) Toledo & Handro, in Hoehne, Relat. Anual Inst. Bot. 1943: 39. 1944. Pachirautiarityi (Hoehne) Hoehne, Indice Bibliogr. Num.: 280. 1951, nom. inval. Rhodagnaphalopsisnitidavar.utiarityi (Hoehne) A. Robyns, Bull. Jard. Bot. État Bruxelles 33: 284. 1963. Type: Brazil. Mato Grosso, Salto do Utiarityi, Rio Papagaio, Apr 1918 (lf, fl), *J.G. Kuhlmann 2145* (lectotype, as “*holotypus*,” designated by Robyns 1963, p. 285: RB barcode 00534522; isolectotypes: R barcode 000027315, R barcode 000027315a, S-PLE-E4219 n.v., SP barcode SP002722).

#### Type.

Brazil. Amazonas, Prope San Gabriel da Cachoeira, ad Rio Negro, Brasiliae borealis, Jan-Aug 1852 [Feb. 1852] (lf, fl), *R. Spruce 2150* (lectotype, designated here: K barcode 000382337 [“São Gabriel, Feb. 1852”; handwritten label]; isolectotypes: B†, BM barcode 000778668, E barcode 00285197, FI barcode 006090, G n.v., K barcode 000382338, LE n.v., M barcode 0211650 [= F neg. no. 19668], NY barcode 00133532, P barcode 05273649 [handwritten label], RB barcode 00060397, TDC [= TCD?] n.v., W barcode 18890017579).

#### Note.

The protologue of *Pachiraobtusa* cites a single collection, “Spruce n. 2150,” which we assume Schumann examined in Berlin (B) and which was subsequently destroyed in WWII. In his revision, Robyns (1963, p. 284) failed to select a lectotype since he made no distinction between duplicates of this collection deposited in various herbaria. We designate here as lectotype of the name a sheet deposited in Kew (K) that has a handwritten locality as well as a narrower collecting date (“Feb. 1852”) than the majority of duplicates that have printed labels and an eight-month range for the collecting date (“Jan.-Aug. 1852”).

In the protologue of *Bombaxpoissonianum*, Schumann (1886) states that he thinks his new species might be the same as the *Pachiranitida* of Decaisne or Kunth (viz., “*Pachiranitida Dcne*.! *Miscell bot*. *1880*. *p. 9*, *an Kunth*?”). *Bombaxpoissonianum*, however, is not a superfluous renaming of *P.nitida* because Schumann (1886, p. 225) included an expression of doubt (see Turland et al. 2018; Art. 52.2, Note 1). There is nothing to tell us who collected the type specimen nor when it was collected. The epithet and author (viz., “*nitida* Kth.”) written on the holotype label appears to be a later addition (the handwriting differs from that of whomever wrote the locality). The specimen was annotated “det. Schumann in Fl. Bras.” and is undoubtedly the one Schumann (1886, p. 225) mentioned in the protologue.

In the protologue of *Bombaxutiarityi*, Hoehne (1927) cites a single collection, “Kuhlmann 2145,” but he does not indicate where it was deposited. When Toledo and Handro in Hoehne (1944, p. 39) proposed a new combination for this species, they mentioned a Kuhlmann specimen housed at SP (as “Inst. Bot. 11.914, leg. J.G. Kuhlmann”), but they did not provide a collection number and failed to use the word “type” or an equivalent (Turland et al. 2018; Art. 7.11). When Robyns (1963, p. 285) proposed a new combination and status for *B.utiarityi*, he also effectively selected a lectotype (Turland et al. 2018; Art. 9.10). The lectotype he designated has an original handwritten label. Inasmuch as *Bombaxutiarityi* agrees with both *B.poissonianum* and *Pachiraobtusa* morphologically and the habitat of occurrence (flooded forest in Amazonia and adjacent Savanna) of all three are the same, we consider *B.utiarityi* to be a synonym of *P.obtusa*.

The designation *Pachirautiarityi* (Hoehne) Hoehne (Hoehne 1951) is not validly published. It was proposed as an alternative for *Bombaxutiarityi*, which was the name Hoehne accepted (see Turland et al. 2018; Art. 36.1(a)).

### ﻿ Pachira paraensis

Taxon classificationPlantaeMalvalesMalvaceae

(Ducke) W.S. Alverson, Novon 4: 7. 1994.

92E3778B-9202-5A4E-B7A0-238FB5EB33CC

Bombax paraense Ducke, Arch. Jard. Bot. Rio de Janeiro 4: 124. 1925. Bombacopsisparaensis (Ducke) A. Robyns, Bull. Jard. Bot. État Bruxelles 33: 213. 1963.

#### Type.

Brazil. Pará, Itaituba, Rio Tapajoz, 26 Aug 1923 (lf, fl), *A. Ducke s.n.* [RB 18094] (lectotype, designated here: RB barcode 00534489; isolectotypes: B† [= F neg. no. 9540], G barcode 00177452, K barcode 000382352, RB barcode 00534497 [without original label], S-R-11283, U barcode 0008391, U barcode 0008392, US barcode 00101944).

#### Note.

Three syntypes are cited in the protologue. Robyns (1963) selected one, “Ducke 18094,” as the lectotype (first step) of *Bombaxparaense* and stated that it was deposited in RB. There are, however, two sheets in RB of this collection. Our lectotypification (second step) narrows Robyns’s choice to a single specimen and it is the same one he annotated as “*lectotypus*.”

### ﻿ Pachira retusa

Taxon classificationPlantaeMalvalesMalvaceae

(Mart.) Fern.Alonso, Revista Acad. Colomb. Ci. Exact. 27(102): 36. 2003.

3BAAB004-84DA-5E3E-AF0F-F937EC629AEA

Fig. 2E, F

Bombax retusum Mart., Flora 8: 28. 1825, Ibid., Nov. Gen. Sp. Pl. 1(4): 92–93, t. 59. 1826 [1824]. Bombacopsisretusa (Mart.) A. Robyns, Bull. Jard. Bot. État Bruxelles 33: 205. 1963.

#### Type.

Brazil. Minas Gerais, In deserto Serro Frio, [1817–20] (fl), *C.F.P. von Martius s.n.* (lectotype, designated here: M barcode 0211681; isolectotype: M barcode 0211682; possible isolectotype: S-PLE-E4218 n.v.).

#### Note.

When Robyns (1963, p. 207) stated that a Martius specimen in Munich (M) was the “*holotypus*” of the name *Bombaxretusum*, he effectively selected a lectotype (Turland et al. 2018; Art. 9.10). However, there are at least two specimens with identical label data in that herbarium that must be considered syntypes as well as a copy of the plate cited in the protologue (“tab. 60,” sphalm. pro 59), but published a year later (Martius 1826). Robyns wrote “*holotypus*” on one of the two specimens and annotated all three elements as “*Bombacopsisretusa* (Mart. et Zucc.) A. Robyns, *comb. nov.*” The ICN (Turland et al. 2018; Art. 7.10) requires that a type designation be effectively published and the mere annotation of a herbarium sheet does not meet this requirement. Our lectotypification (second step; see Turland et al. 2018; Art. 9.17) narrows Robyns’ selection to the specimen with the best flowering material.

Robyns (1963, p. 205) attributed the basionym to “Mart. et Zucc.,” but according to Stafleu and Cowan (1981, p. 329) authorship is to be attributed to von Martius alone. Fernández-Alonso (2003, p. 36) also included J.G. Zuccarini as a co-author of this species name. Interestingly, Schumann (1886, p. 226) recognized “*Bombaxretusum* Mart.” while citing “*Bombaxretusum Mart*. *et Zucc*.! *Nov. gen. et spec. I. 92. t. 59*.” The article that includes the validating description of *B.retusum* (Martius 1825) clearly states that Martius collected and described (“collegit et descriptsit”) the plants while Zuccarini organized the plates, etc. (“*Pingendas curavit et secundum auctoris schedulas digessit*”).

### ﻿ Pachira sordida

Taxon classificationPlantaeMalvalesMalvaceae

(R.E. Schult.) W.S. Alverson, Novon 4: 8. 1994.

5BB6F9BB-9E3E-5DD5-85F2-0CC20A1B1FA8

Bombax sordidum R.E. Schult., Bot. Mus. Leafl. 16: 75. 1953. Rhodognaphalopsiscoriaceavar.sordida (R.E. Schult.) A. Robyns, Bull. Jard. Bot. État Bruxelles 63: 292. 1963. Pochotasordida (R.E. Schult.) Steyerm. & W.D. Stevens, Ann. Missouri Bot. Gard. 75: 398. 1988.

#### Type.

Colombia. Vaupés, Río Negro, San Felipe (El Castillo), below confluence of Ríos Guainía and Casiquiare, 12 Dec 1947 (lf, fr), *R.E. Schultes & F. López 9342* (lectotype, designated here: GH barcode 00066501; isolectotypes: GH barcode 00066502, GH barcode 00066503).

#### Note.

A single collection, “R.E. Schultes & F. López 9342,” is cited in the protologue of *Bombaxsordidum*. When Robyns (1963, p. 292) proposed a new combination and status for this name, he indicated that the holotype was deposited in the Gray Herbarium: “Colombie: Vaupes: *Schultes & F. López*, *9342* (f., fr., *holotypus* GH)”. There are, however, three sheets of this collection in that herbarium, and although one was annotated as the holotype by Robyns, the ICN (Turland et al. 2018; Art. 7.10) requires that a type designation be effectively published. The mere annotation of a herbarium sheet does not meet this requirement, hence our lectotypification (second step). We designate here the specimen that has the greatest abundance of indumentum on the abaxial surfaces of the leaflets because this character is considered diagnostic by Robyns (1963, p. 292, viz. “*a specie foliolorum lamina infra densissime et sordide pulveraceo-lepidota sat differt*”).

## Supplementary Material

XML Treatment for Pachira aquatica

XML Treatment for Pachira calophylla

XML Treatment for Pachira duckei

XML Treatment for Pachira endecaphylla

XML Treatment for Pachira glabra

XML Treatment for Pachira humilis

XML Treatment for Pachira insignis

XML Treatment for Pachira macrocalyx

XML Treatment for Pachira minor

XML Treatment for Pachira nervosa

XML Treatment for Pachira obtusa

XML Treatment for Pachira paraensis

XML Treatment for Pachira retusa

XML Treatment for Pachira sordida
